# Optical fiber meta-tips

**DOI:** 10.1038/lsa.2016.226

**Published:** 2017-03-10

**Authors:** Maria Principe, Marco Consales, Alberto Micco, Alessio Crescitelli, Giuseppe Castaldi, Emanuela Esposito, Vera La Ferrara, Antonello Cutolo, Vincenzo Galdi, Andrea Cusano

**Affiliations:** 1 Optoelectronic Division, Department of Engineering, University of Sannio, I-82100 Benevento, Italy; 2 Waves Group, Department of Engineering, University of Sannio, I-82100 Benevento, Italy; 3 Institute for Microelectronics and Microsystems, National Research Council, I-80131 Napoli, Italy; 4 UTTP-MDB, Materials and Devices, ENEA—Portici Research Center, I-80055 Portici, Napoli, Italy

**Keywords:** Fiber optics, metasurfaces, plasmonics, wavefront manipulation

## Abstract

We report on the first demonstration of a proof-of-principle optical fiber ‘meta-tip’, which integrates a phase-gradient plasmonic metasurface on the fiber tip. For illustration and validation purposes, we present numerical and experimental results pertaining to various prototypes implementing generalized forms of the Snell’s transmission/reflection laws at near-infrared wavelengths. In particular, we demonstrate several examples of beam steering and coupling with surface waves, in fairly good agreement with theory. Our results constitute a first step toward the integration of unprecedented (metasurface-enabled) light-manipulation capabilities in optical-fiber technology. By further enriching the emergent ‘lab-on-fiber’ framework, this may pave the way for the widespread diffusion of optical metasurfaces in real-world applications to communications, signal processing, imaging and sensing.

## Introduction

Metamaterials are artificial composites, which attain their distinctive properties from a careful structural arrangement of dielectric and/or metallic subwavelength-sized constituents, rather than their chemical composition^1^. Over the past 15 years, they have received an exponentially growing interest in many scientific and engineering fields, as a possible route to achieve unconventional light-matter interaction effects (such as ‘negative’ refraction^2^ and ‘superlensing’^3^), as well as unprecedented field-manipulation capabilities via proper spatial tailoring of the constitutive parameters^4^. In spite of such extremely promising prospects, the practical applications of metamaterials to optics and photonics remain limited, mainly due to the significant technological challenges posed by the fabrication process of three-dimensional (3-D) bulk nanostructures^5, 6, 7^. This has generated a surge of interest in 2-D implementations (‘metasurfaces’), thanks to the easier fabrication as well as on-chip integrability.

Similar to reflect-arrays and transmit-arrays at radio-frequency^8, 9^, optical metasurfaces exploit 2-D arrays of resonating elements to spatially tailor the phase and amplitude distributions of an incident wave field. However, unlike radio-frequency implementations, optical implementations can exhibit a deeply-subwavelength profile, by relying, e.g., on plasmonic^10^ or dielectric elements^11^. Stimulated by the groundbreaking studies by Yu *et al.*^12^, which demonstrated the capabilities of metasurfaces based on V-shaped plasmonic nanoantennas in terms of anomalous reflection/refraction and wavefront shaping, several research groups have explored and proposed various alternative designs^13, 14, 15, 16, 17, 18, 19, 20, 21, 22, 23, 24^. Currently, ‘flat’ optics and photonics^25, 26, 27^ constitute a very promising research thrust, with a plethora of potential applications to various fields, ranging from imaging to computing^28, 29, 30, 31, 32, 33^.

As highlighted in recent influential review papers^27, 34^, among the possible developments, it appears of particular strategic importance the integration of metasurfaces with fiber-optics technology. Indeed, as this technology has established itself in communications systems and has recently expanded to sensing applications, the increasing demand for better performance and advanced functionalities has led to the exploration of new fabrication strategies and concepts. Within this framework, the emergent ‘lab-on-fiber’ paradigm^35, 36, 37^ envisages fiber-optics platforms integrated with nanostructured photonic and/or plasmonic materials capable of controlling the light at the nanoscale. This represents a very promising pathway to novel ‘all-in-fiber’ multifunctional nanoprobes hosting ultracompact labs, which can disruptively enlarge the conventional fiber-optics functionalities, and may find a broad variety of applications including optical processing, environmental and life science, homeland security and so on^37^. To deal with unusual substrates such as an optical-fiber tip, several fabrication processes have been developed^36^, leading to the realization of multifunctional and multi-responsive nanoprobes for applications including optical-fiber tweezers^38, 39, 40^, *in vivo* single molecule imaging^41, 42^, scanning near-field optical microscopy^43, 44, 45, 46^, fiber top cantilevers^47^ and sensing^48, 49, 50, 51^.

Within the lab-on-fiber framework, the integration of metasurfaces would constitute a crucial step forward, as it would provide unprecedented light-manipulation capabilities. Likewise, the technological maturity and broad diffusion of fiber optics in real-world applications may substantially boost the practical applicability of optical metasurfaces.

Here, we show that phase-gradient plasmonic metasurfaces can be successfully fabricated on an optical-fiber tip. As a proof-of-concept of these ‘meta-tips’ (MTs), we design and fabricate several prototypes that implement the steering of an impinging beam in desired directions. Moreover, with a view toward sensing applications, we exploit this mechanism to excite surface waves, whose interplay with the nanoantenna resonances may provide further degrees of freedom to enhance the sensitivity of plasmonic nano-arrays.

## Materials and methods

### Idea and geometry

**Figure 1:**
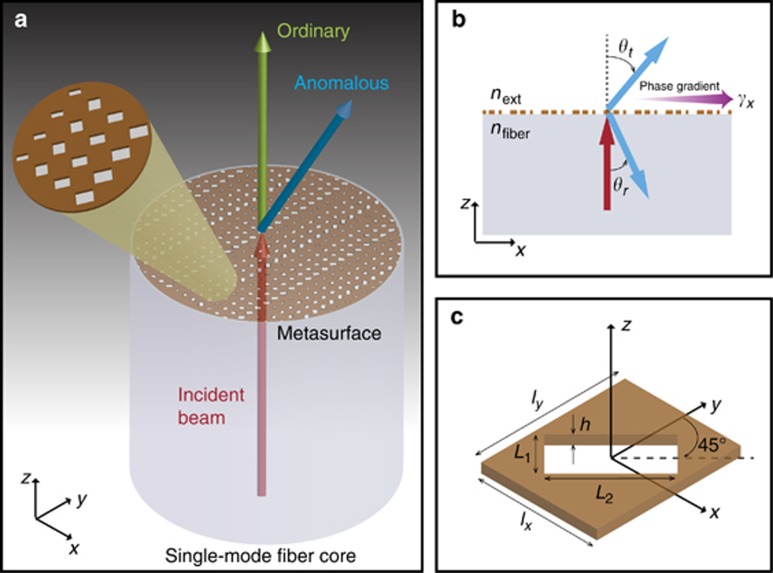
Illustration of the idea and geometry. (**a**) Pictorial sketch (not in scale) of an optical-fiber MT. A plasmonic metasurface (with details magnified in the inset) is laid on the tip of an optical fiber, covering the entire core region. The metasurface impresses a linear-phase profile (constant gradient, along the *x*-direction) in the wavefront of a given component of an impinging beam. This yields splitting in the transmitted beam, with an ordinary (co-polarized) component propagating along the incidence direction, and an anomalous component (with generally different polarization) undergoing a phase-gradient-induced steering of an angle. A similar phenomenon (not shown for better visibility) occurs in reflection as well. (**b**) Illustration of the generalized Snell's refraction/reflection laws in Equation (1). (**c**) Geometry (not in scale) of the unit cell: a rectangular nanohole (rotated of 45° in the *x* – *y* plane) milled in a gold layer.

The basic idea underlying our MT design is outlined in Figure 1a. We consider the tip facet of an optical fiber covered (over the entire core region) by a plasmonic metasurface, which impresses a desired phase profile in a suitably-polarized field component. As an example, in the present study we assume a linear-phase distribution along the *x*-direction. Accordingly, a light beam normally incident from the core region will undergo a splitting in transmission (and reflection, not shown in Figure 1a schematic), with: (i) an ordinary component experiencing no phase-gradient; and (ii) the emergence of an anomalous beam (with generally different polarization) steered of an angle along the *x*-direction. Such polarization-conversion mechanism is inherent of single-layer implementations of plasmonic metasurfaces, as a device to attain a 2*π* phase span in the transmitted field^26^. As schematically illustrated in Figure 1b for the specific case of interest (normal incidence), the phenomenon can be modeled via generalized Snell’s laws^12^,


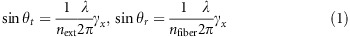


which relate the steering angles in transmission (*θ*_t_) and reflection (*θ*_r_) to the metasurface-induced phase-gradient *γ*_*x*_, with *n*_fiber_ and *n*_ext_ denoting the refractive indexes of the fiber and external regions, respectively, and *λ* the vacuum wavelength. Clearly, in the absence of phase-gradient (*γ*_*x*_=0), Equations (1) trivially reduce to the conventional Snell’s laws. Our metasurface design, inspired by Babinet’s principle, features an ‘inverted’ configuration obtained by patterning a 50 nm gold layer with rectangular nanoholes of variable size. Besides being particularly suited to focused-ion-beam (FIB) nanofabrication processes, this inverted design has been shown to provide a higher polarization efficiency than the more conventional (nanopatch) configuration^17^. The unit-cell geometry (Figure 1c) basically comprises a rectangular nanohole rotated by 45° in the *x*−*y* plane.

### Design procedure

The design of Babinet-inverted plasmonic metasurfaces has been discussed in Ni *et al.*^17^. It is well known that the optical properties of periodically nanopatterned-metal layers generally depend on the complex interplay between a surface plasmon polariton (which can couple to the normally impinging light in view of the array-induced phase-matching mechanism) and the localized waveguide modes supported by the nanoholes^52^. As shown in Ni *et al.*^17^, by properly tuning the nanohole sidelengths *L*_1_ and *L*_2_ so as to work in a neighborhood of such resonance frequency, an arbitrary phase (within the full 2*π* range) can be impressed in the transmitted/reflected components with suitable polarization. More specifically, as a consequence of the nanohole symmetry (Figure 1c), the local transmission coefficient of the cross-polarized component is identical for normal plane-wave incidence with *x*- or *y*-polarized electric field. As anticipated, this implies that a normally-incident illumination with linearly-polarized electric field forming an angle *α* with the *x*-axis yields two transmitted beams (see the pictorial sketch in Figure 1a): an ordinary one, co-polarized, and (ideally) experiencing zero phase-gradient; and an anomalous one, with polarization direction rotated by an angle (90°−*α*), and experiencing the steering effect impressed by the metasurface^26^. The two beams are co-polarized for illumination polarized along the symmetry axis *x*=*y* (*α*=45°), and orthogonally polarized for *x*- or *y*-polarized illumination (i.e., *α*=0 or *α*=90°, respectively). In our design procedure, we first compute (numerically) the co- and cross-polar transmission coefficients of a 2-D periodic array (with *l*_*x*_=*l*_*y*_=1 μm) made of identical nanoholes. More specifically, assuming *x*-polarized plane-wave excitation at *λ*=1.56 μm, we consider moderate variations of *L*_1_ and *L*_2_ around their resonance values (which can be roughly estimated via simple analytical models^53^), so as to generate some ‘look-up’ maps, shown in Supplementary Fig. S1, which directly relate the nanohole dimensions to the cross-polar transmission-coefficient phase.

As a proof-of-concept, we realized via FIB milling five MT prototypes, hereafter indicated as MT_*m*_ (*m*=1,…,5). More specifically, at the telecom wavelength *λ*=1.56 μm, we implemented various representative values of the phase-gradient *γ*_*x*_, whereas maintaining a constant phase distribution along *y*. These phase distributions are synthesized via a ‘supercell’ comprising *N* unit cells as in Figure 1c, arranged along the *x*-direction with period *l*_*x*_, and suitably modulated dimensions *L*_1_ and *L*_2_. In particular, by exploiting the previously computed look-up maps, these dimensions are chosen in such a way an incremental phase difference 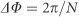 is attained in the cross-polar transmission and reflection coefficients between neighbor unit cells. Replication of such ‘supercell’ along *x* (with period 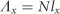) and *y* (with period 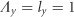 μm) yields the desired phase distribution, with gradient 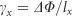. Further details on the design procedure are provided in the Supplementary Information.

Table 1 summarizes the main parameters of the five designs. For the MT_1_–MT_4_ designs, which implement the beam steering, the nominal transmission angles *θ*_*t*_ of the anomalous beams are indicated. The MT_5_ design, instead, implements the excitation of a surface wave. For all designs, only the sidelengths *L*_1_ and *L*_2_ of the nanoholes in the first half of the supercell are explicitly given; the elements in the second half are obtained by a rotation of 90° in the *x*–*y* plane, which provides a *π* phase shift in the cross-polar scattering parameters.^26^

### Numerical modeling

For the metasurface design, as well as for the computation of the far-field profiles and the field maps shown hereafter, we rely on the finite-element software package COMSOL Multiphysics (www.comsol.com). For cross-validation, as well as for the computation of reflectivity spectra, we utilize a 2-D version of the rigorous-coupled-wave-analysis^54^ (RCWA) implemented in a public-domain numerical code (www.sourceforge.net/projects/rcwa-2d/files/). In all simulations, we consider a standard dispersion model for gold^55^, and nondispersive, lossless models for silica (optical fiber) and SiO_*x*_ (overlay for surface-sensitivity characterization), with refractive indexes 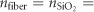 1.45 and 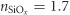, respectively. The external region is assumed as air (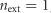). Further details can be found in the Supplementary Information.

### Prototype fabrication

We start from a Corning SMF-28 single-mode fiber (with core and cladding diameters of 8 and 125 μm, respectively), which is cleaved to obtain a smooth surface. A segment (of about 10 mm) of the fiber is first subjected to ethanol rinsing, and subsequently positioned on an apposite sample holder. The fiber tip is coated with a 50 nm gold layer via electron beam evaporation (Kenosistec CL400C, Binasco (MI), Italy), with adhesion enhanced by a 2 nm intermediate chrome layer. A FIB instrument (Quanta 200 3D FEI, Hillsboro, OR, USA) is used to pattern the gold layer, by using 50 pA beam current and 30 kV accelerating voltage. The desired pattern is milled (with 5000 × magnification) by rastering the ion beam via parallel writing strategy, and employing an input text file where all the rectangular holes are defined in terms of size, spatial coordinates and rotation angles. Results from the morphological characterization of the samples can be found in Supplementary Fig. S3 and Supplementary Table S1.

It is worth stressing that the patterned areas (Table 1) are dimensioned so as to cover a substantial part of the fiber mode region. It can readily be estimated that at the edges of these areas the illuminating field profile (approximately a Gaussian beam with waist size of 5 μm) has decayed at least 24 dB below its peak value, and is therefore effectively negligible. This is also consistent with experimental indications from previous lab-on-fiber studies^56^.

As previously mentioned, four prototypes (MT_1_–MT_4_) implement the beam steering with various angles. On the other hand, as it will be clear hereafter, the MT_5_ prototype (as well as a phase-gradient-free benchmark) is designed having in mind sensing applications. Within this framework, to characterize its surface sensitivity, the sample is placed inside a very high frequency plasma enhanced chemical vapor deposition (PECVD) chamber using a suitable holder that locates it in the correct position. A thin SiO_*x*_ layer is deposited by using an ultra-high vacuum cluster tool deposition system (MVSystems Inc., Golden, CO, USA). The fiber tip is placed at a distance less than 15 mm from the electrode. The process is carried out at 150° temperature, 2.5 Torr pressure and 6 W power. Pure silane, hydrogen and carbon dioxide are used, with a deposition rate of about 1.88 Å s^−1^. Via an atomic-force-microscope measurement, an overlay thickness value of about 40 nm is verified (see Supplementary Information for details).

### Experimental characterization

The far-field characterization of the fabricated MT samples implementing the beam steering is carried out by means of an experimental setup relying on an infrared vidicon camera (see the schematic in Supplementary Fig. S4). Precise and repeatable positioning of different samples is ensured by the use of an ad-hoc plastic holder for the fiber MT. The sample end-face is positioned in the vicinity of the camera by means of a compact 3-axis micrometer positioning system. Precise control of the MT distance from the camera receiving window (i.e., the input glass face of the vidicon tube) is allowed by a position reference mounted onto the plastic holder. The fiber MT is illuminated with a narrowband laser source (centered at *λ*=1.56 μm) by means of a tunable laser (Yokogawa/Ando AQ4321A, Tokyo, Japan), and the transmitted far-field is collected by the vidicon camera (Hamamatsu C2741-03 camera head + C2471 camera controller; www.hamamatsu.com). The camera head is shielded from visible light to improve the optical quality of the digital images (786 × 576 pixels, 256 gray levels), which are acquired via a PCI-1407 IMAQ acquisition board, sent to a display and transferred to a personal computer for post-processing. In particular, the field-intensity maps shown hereafter are measured by positioning the MT at a distance of 4 mm from the receiving window of the camera; taking into account the distance between this latter and the photoconducting target inside the vidicon tube (4 mm), a total distance of 8 mm is estimated between the MT and the target plane. The transmission angles are estimated via a differential measurement scheme (Supplementary Fig. S5). For the polarization measurements, a fiber polarization controller (Thorlabs FPC560, Newton, NJ, USA) is connected at the output of the tunable laser, and a linear polarizer (Thorlabs LPIREA100-C, Newton, NJ, USA) is mounted on a continuous rotation mount (Thorlabs CRM1/M, Newton, NJ, USA) positioned right before the vidicon camera (see Supplementary Information for more details). In this case, a distance of 5.9 mm is estimated between the MT and the target plane.

For the surface-sensitivity characterization of the MT_5_ prototype (and its phase-gradient-free benchmark), a standard reflection setup (schematized in Supplementary Fig. S6) is utilized. The fiber MT is illuminated by means of a supercontinuum light source (covering the wavelength range 1.1–2.4 μm), whereas a 2 × 1 directional coupler is used to redirect the reflected signal to an optical spectrum analyzer (Yokogawa/Ando AQ6317C, having a wavelength range 600–1750 nm). The acquired spectrum is transferred (via a general purpose interface bus connection) to a personal computer for post-processing. Further details are provided in the Supplementary Information.

## Results and discussion

### Beam steering

**Figure 2:**
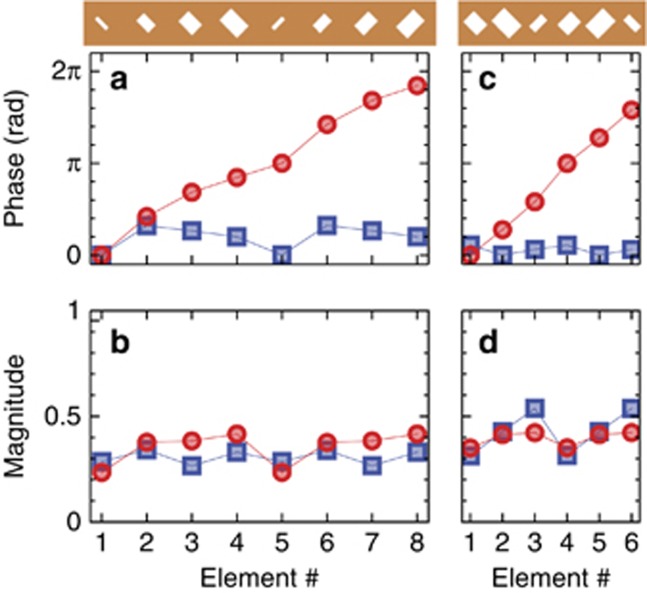
Results from the design procedure (MT_1_ and MT_3_). (**a**, **b**) Numerically-synthesized phase and magnitude distributions, respectively, of the transmission coefficient pertaining to the single nanoholes in the supercell (shown on top) of the MT_1_ design (with parameters as given in Table 1), for the co-polarized (blue square markers) and cross-polarized (red circle markers) components, assuming an infinite periodic array of period *l*_x_ = *l*_y_ = 1 μm, under normally-incident *x*-polarized plane-wave illumination at *λ* = 1.56 μm. Element #1 is chosen as phase reference. Continuous curves are guides to the eye only. (**c**, **d**) Same as above, but for MT_3_ design.

With specific reference to the MT_1_ and MT_3_ designs, Figure 2 shows the numerically-synthesized magnitude and phase profiles of the transmission coefficients (for normally-incident *x*-polarized illumination) over a supercell. Approximately linear-phase distributions (with different gradients) are observed for the cross-polarized components, whereas the co-polar phase as well as the two magnitude distributions remain more or less uniform. The corresponding distributions for the MT_2_ and MT_4_ design are shown in Supplementary Fig. S2.

**Figure 3:**
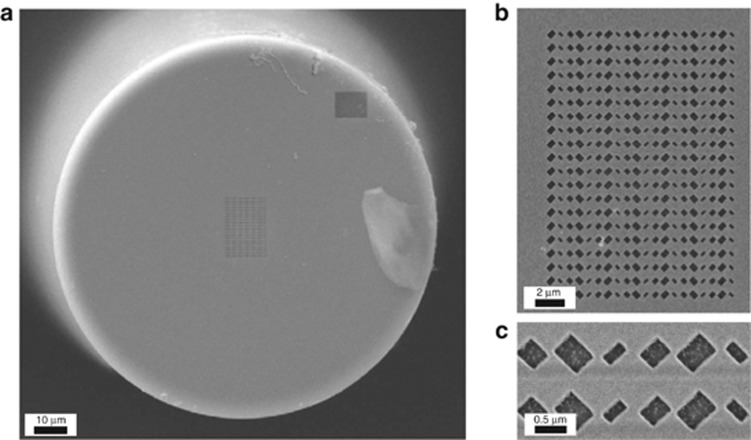
Example of a fabricated sample. (**a**) SEM image of the MT_3_ sample, displaying the entire fiber cross-section. (**b**, **c**) Two magnified details, showing the entire metasurface and two unit cells, respectively.

Figure 3 displays some scanning-electron-microscope (SEM) images of the MT_3_ prototype, including the fiber tip and two magnified details of the metasurface.

**Figure 4:**
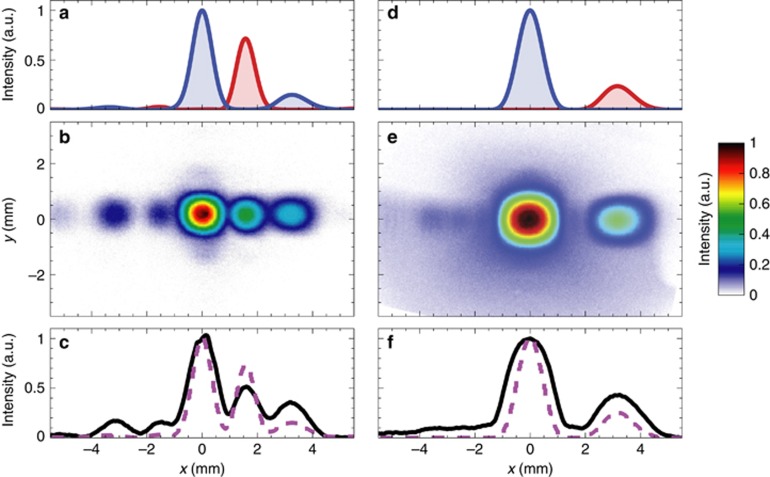
Far-field characterization (MT_1_ and MT_3_) without polarization control. (**a**) Simulated electric field-intensity profiles (at *z* = 8 mm and *y*=0) of the ordinary and anomalous beams (blue and red curves, respectively), for MT_1_ sample (with parameters as given in Table 1). Results are obtained by averaging the co-polar and cross-polar responses, respectively, under normally-incident *x*- and *y*-polarized illuminations at *λ* = 1.56 μm. (**b**) Measured field-intensity map at *z* = 8 mm. (**c**) Transverse cuts at *y* = 0 comparing the measured (black-solid curve) and simulated (magenta-dashed curve) results. Numerical results are obtained by averaging the total electric-field intensities for normally-incident *x*- and *y*-polarized illuminations. The structure is considered as infinitely-periodic along *y*, whereas, along the *x*-direction, a finite-size ∼20 μm is assumed, together with a Gaussian-beam taper (with waist size of 5μm) in the illumination. (**d**–**f**) Same as above, but for MT_3_ sample.

With reference to the MT_1_ and MT_3_ samples, Figure 4 summarizes the far-field characterization at the operational wavelength *λ*=1.56 μm, without direct polarization control of the incident and transmitted fields. More specifically, Figure 4b and 4e show the measured field-intensity maps at 8 mm from the MT samples. As a reference, Figure 4a and 4d show the simulated co-polar and cross-polar (blue and red curves, respectively) intensity profiles at *z*=8 mm and *y*=0, averaged over the *x*- and *y*-polarized illuminations. In spite of the lack of polarization control, the comparison between the numerical profiles and the measured intensity maps still allows a clear-cut interpretation of the main and secondary peaks in terms of the ordinary (co-polarized) and anomalous beams, respectively. Overall, as shown in Figure 4c and 4f, a fairly good agreement is observed between the measured intensity profiles (black-solid curves) and corresponding simulations (magenta-dashed curves). The anomalous refraction angles estimated from the experimental data are 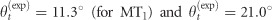 (for MT_3_), in very good accord with the theoretical estimates from Equation (1) (given in Table 1).

The numerical results also correctly reproduce some secondary side lobes in the ordinary and anomalous beams, especially visible in the MT_1_ case, which can be attributed to slight nonlinearities in the designed phase profiles (see, e.g., Figure 2a) as well as truncation effects.

**Figure 5:**
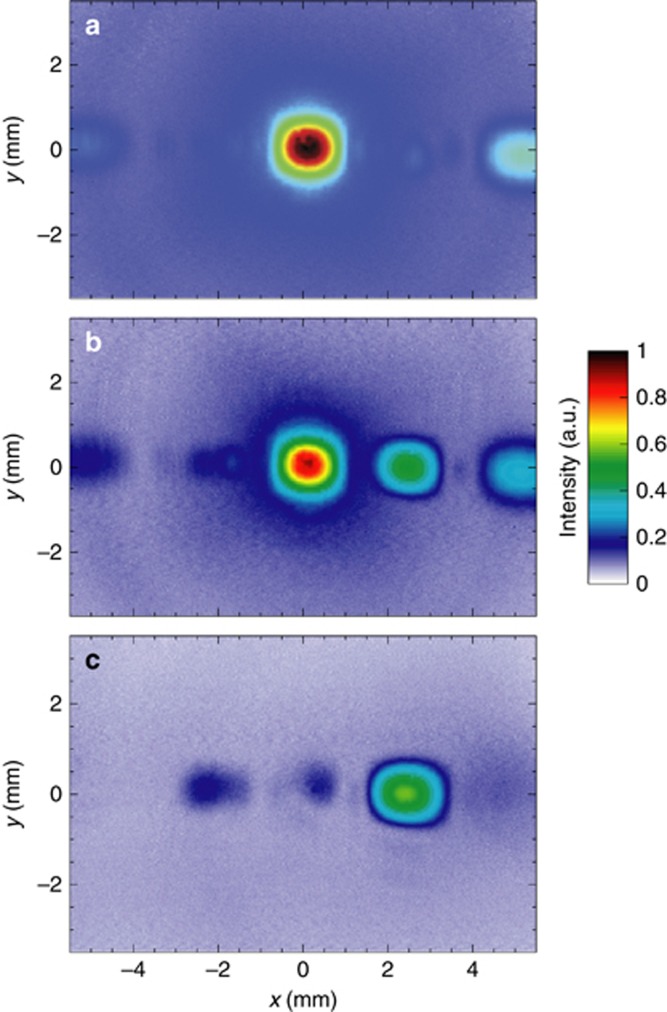
Far-field characterization (MT_3_) with polarization control. (**a**–**c**) Measured field-intensity maps at *z* = 5.9 mm for MT_3_ sample (with parameters as given in Table 1) pertaining to the *y*-, oblique (45°) and *x*-polarized components, respectively, and assuming a *y*-polarized incident field (see also Supplementary Movie 1 for a finer sampling of the selected transmitted polarization).

As an ultimate validation of the phenomenon, we also carried out polarization measurements (see Supplementary Information for details). With reference to the MT_3_ sample, Figure 5 shows the far-field maps (at *z*=5.9 mm) assuming a *y*-polarized incident field, and selecting three representative linear polarization states of the transmitted field. The assumed incident polarization yields a particularly clear-cut difference between the ordinary and anomalous beams, which are co- and cross-polarized, respectively. Accordingly, Figure 5a shows the *y*-polarized field map, where a main peak is clearly observed, representative of the (co-polarized) ordinary beam. As also observed in the previous measurements (without polarization control), the peripheral minor peak is attributable to nonidealities. Figure 5c shows instead the *x*-polarized field map, where the ordinary beam (and the peripheral one) disappears, and a different peak appears, representative of the (cross-polarized) anomalous beam. For an intermediate case, pertaining to a 45° oblique polarization (Figure 5b), both peaks are correctly observed. Supplementary Movie 1 shows the evolution of the measured field map with a finer sampling (5°) of the selected polarization state in transmission, from which it can be observed the gradual disappearing of the ordinary beam and the appearing of the anomalous one.

Discrepancies between experimental and numerical results are mainly attributable to fabrication tolerances in the gold-layer thickness as well as in the dimensions of the nanoholes (see the Supplementary Information for the morphological characterization). Other potential sources of uncertainty are associated with the FIB milling process, which is known to induce a doping of the fiber glass^57^, and hence unmodeled shifts in the resonance wavelength of the nanoholes. Nevertheless, our study indicates that the proposed designs are quite robust with respect to the above fabrication-related effects.

Qualitatively similar results are observed for the other two beam-steering design prototypes MT_2_ and MT_4_, as shown in Supplementary Fig. S7.

We also estimated numerically the efficiency, in terms of the fraction of the impinging power that gets transferred to the anomalous beam (see Supplementary Information for details). As summarized in Table 2 for the four beam-steering prototypes, values ranging within 7–12% are obtained. These results are essentially in line with the 10% figures observed in previous studies on Babinet-inverted plasmonic metasurfaces in planar technology^17^. Nevertheless, there are several alternatives (e.g., Huygens’ dielectric metasurfaces^23^) that can provide much higher efficiencies.

Moreover, we highlight that, although the general character of the underlying resonance phenomenon is not narrowband, our investigation was inherently restricted within the narrow spectral operational range (1520–1620 nm) of the tunable laser source utilized. Within this wavelength range, we observed negligible variations of the efficiency, and variations up to approximately 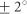 in the anomalous beam-steering angle. These values are larger than our estimated measurement uncertainty (see Supplementary Information for details), and are in line with the theoretical predictions from Equation (1) (with the phase-gradient *γ*_x_ assumed as constant).

It is worth pointing out that, via suitably large values of *γ*_x_, it is possible to drive the anomalous transmitted beam in the evanescent range, so as to couple it with a surface wave that propagates along the *x*-direction at the MT interface, and is exponentially bound along *z*^58, 59^. In what follows, we explore possible applications of this mechanism to sensing scenarios.

### Perspectives in sensing applications

During the last decade, plasmonic nanosensors have established themselves as advanced tools for biosensing applications. A broad variety of configurations have been proposed, and the field is fast-paced and growing. A common aspect of the ongoing research efforts remains the continuous quest for improved sensitivity^60, 61^, and different strategies have been so far adopted, mainly relying on the proper choice of the nanostructure geometry and dimension, plasmon coupling effects and amplification mechanisms based on nanoparticle growth^62^.

One might wonder to what extent the aforementioned mechanism of surface-wave excitation mediated by the phase-gradient could provide further degrees of freedom to tailor the plasmonic sensitivity of our MTs. To this aim, we investigated (both theoretically and experimentally) the possibility to exploit such mechanism to enhance the light-matter interaction and, hence, to provide an increased sensitivity to local refractive index changes with respect to standard (phase-gradient-free) plasmonic benchmarks.

**Figure 6:**
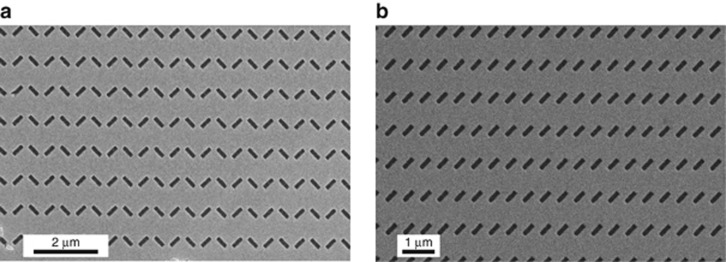
Examples of fabricated samples. (**a**, **b**) SEM images (magnified details) of the MT_5_ and benchmark (phase-gradient-free) samples, respectively.

**Figure 7:**
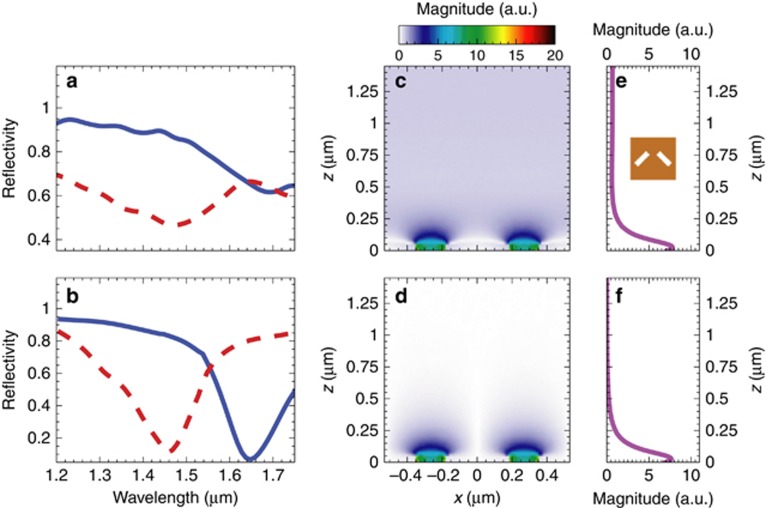
Perspectives in sensing applications (MT_5_). (**a**, **b**) Measured and simulated, respectively, reflectivity spectra in the absence (red-dashed curves) and presence (blue-solid curves) of a 40 nm overlay of SiO_*x*_. (**c**, **d**) Simulated electric-field magnitude map over a supercell (at *y*=0, nearby the MT) for the co-polarized and cross-polarized component, respectively, at *λ* = 1.46 μm, assuming a normally-incident *x*-polarized plane-wave illumination. (**e**, **f**) Corresponding longitudinal cuts at *x* = 0.265 μm. The inset shows the MT_5_ supercell (with parameters as given in Table 1). Fields are normalized with respect to the incident-field amplitude.

Within this framework, the implied high values of phase-gradient can be attained in our configuration by reducing the number of nanoholes per unit cell and/or by increasing the phase difference ***ΔΦ*** between neighbor elements. In our implementation (MT_5_ design in Table 1), we consider ***ΔΦ***=π (i.e., the maximum value that still guarantees the correct reconstruction of the linear-phase profile), and *l*_x_=0.53 μm (which ensures that the anomalous reflected beam too is driven in the evanescent range). Figure 6a shows a SEM image of the fabricated prototype. The resulting supercell (see also the inset in Figure 7c) is reduced to two identical nanoholes, rotated of 90° in the *x–y* plane; the underlying symmetry implies that the excited surface waves travel along both ±*x*-directions^63^. Such configuration is particularly interesting as it admits a simple phase-gradient-free counterpart (which will be used as a benchmark to compare the sensing performance), thereby allowing some insightful considerations on the effects of the phase-gradient, as discussed hereafter.

Figure 7a and 7b show (red-dashed curves) the measured and simulated reflectivity spectra, respectively, pertaining to the MT_5_ sample; they both exhibit a resonant dip centered around *λ*=1.47 μm (*λ*=1.46 μm in the simulated spectrum), and are in quite good agreement both from the qualitative and quantitative viewpoints. To gain some insight in the nature of the observed resonance, Figure 7c and 7d show the simulated field maps (in the *x* – *z* plane, nearby the MT interface) at the resonant wavelength, for *x*-polarized plane-wave illumination. A local field-enhancement, attributable to the resonance of the single nanoholes, can be observed in both the co-polarized and cross-polarized components. However, the transmitted co-polarized field (not affected by the metasurface phase-gradient) shows a propagating character, whereas the cross-polarized field clearly exhibits the distinctive features of a surface wave. This is more evident in the corresponding field-cuts (along the *z*-direction) shown in Figure 7e and 7f, respectively.

**Figure 8:**
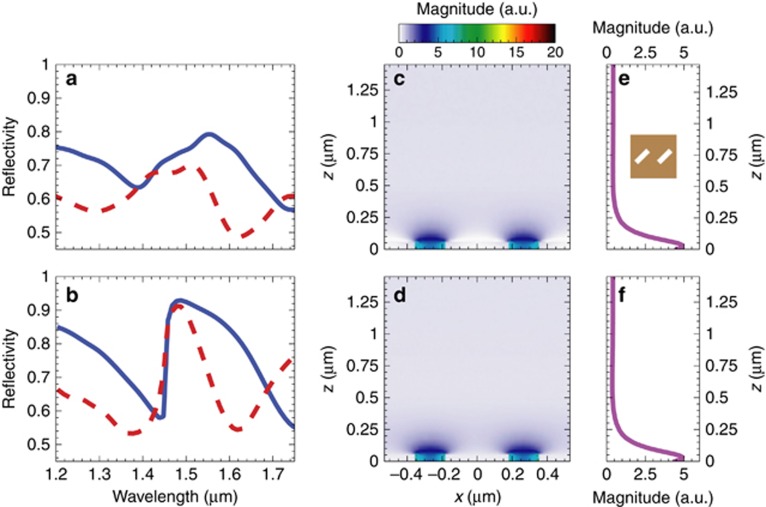
Perspectives in sensing applications (benchmark MT). (**a**, **b**) Measured and simulated, respectively, reflectivity spectra in the absence (red-dashed curves) and presence (blue-solid curves) of a 40-nm-thick overlay of SiO_*x*_. (**c**, **d**) Simulated electric-field magnitude map over a supercell (at *y*=0, nearby the MT) for the co-polarized and cross-polarized component, respectively, at *λ* = 1.62 μm, assuming a normally-incident *x*-polarized plane-wave illumination. (**e**, **f**) Corresponding longitudinal cuts at *x* = 0.265 μm. The inset shows the benchmark supercell. Fields are normalized with respect to the incident-field amplitude.

As a meaningful benchmark configuration (see the SEM image in Figure 6b and the supercell in the inset of Figure 8e), we selected a periodic metasurface featuring the same nanoholes as in the MT_5_ configuration, but without the 90° rotation. In this way, the phase-gradient effects are removed, whereas the resonant effects related to the rectangular nanoholes are preserved. By comparing the sensing responses of the MT_5_ and benchmark design, we can therefore highlight the possible effects of the phase-gradient.

The measured and simulated reflectivity spectra of the benchmark sample (shown in Figure 8a and 8b, respectively) exhibit a resonant dip centered at a higher wavelength (*λ*=1.62 μm) than the previous case. Once again, measurements and simulations agree fairly well. From the simulated field maps at the resonant wavelength (Figure 8c and 8d), similarly to the phase-gradient MT_5_ case (cf. Figure 7c and 7d), we observe a local field-enhancement attributable to the resonance of the nanohole (chosen as identical in the two designs). However, an important difference is that now both the co-polarized and cross-polarized transmitted components exhibit propagating characteristics; this clearly highlights the impact of the phase-gradient entailed by the MT_5_ design.

**Figure 9:**
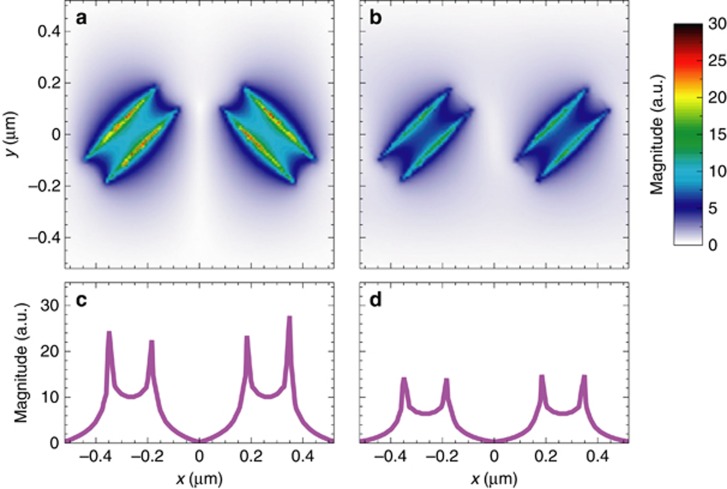
Resonant field distributions (MT_5_ and benchmark). (**a**, **b**) Simulated electric-field magnitude maps over a supercell at the MT interface *z* = 50nm, for the MT_5_ (at *λ* = 1.46 μm) and benchmark (at *λ* = 1.62 μm) configurations, respectively, assuming a normally-incident *x*-polarized plane-wave illumination. (**c**, **d**) Corresponding transverse cuts at *y*=0. Fields are normalized with respect to the incident-field amplitude.

Another important aspect worth exploring is the effect of the phase-gradient in establishing the local field distribution occurring at the MT surface. To this aim, Figure 9 compares the two resonant field distributions occurring at the MT interface. From the field maps (Figure 9a and 9b) and corresponding cuts (Figure 9c and 9d), it is evident that the MT_5_ configuration exhibits a sensibly higher field-enhancement at the interface, which is crucial in determining the surface sensitivity to local refractive index variations^30^.

Having in mind label-free chemical and biological sensing applications, we evaluate (and compare) the surface sensitivity of both the phase-gradient MT and its gradient-free benchmark, by considering the resonance wavelength-shift produced by the deposition of a nanosized dielectric overlay^56, 64^. Accordingly, we repeat the reflectivity measurements of both samples after the deposition of a 40 nm SiO_*x*_ overlay (refractive index 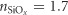, see Supplementary Information for details). For a more direct comparison with the responses in the absence of the overlay, these new measured spectra are also shown (blue-solid curves) in Figures 7a and 8a. A higher redshift (224 nm) is observed for the phase-gradient MT_5_ sample when compared with the gradient-free benchmark (132 nm). This is also consistent with the numerical predictions (blue-solid curves in Figures 7b and 8b).

This first and interesting observation opens up intriguing perspectives in the exploitation of phase- gradient as a further degree of freedom in determining and tailoring the surface sensitivity of plasmonic nanostructures. However, further in-depth investigations are required to ascertain the role and interplay of the various phase-gradient-induced phenomena (surface wave, field enhancement) in the observed surface-sensitivity enhancement.

## Conclusions

To sum up, we have demonstrated a proof-of-concept MT that integrates a phase-gradient plasmonic metasurface on an optical-fiber tip. Our results represent a first, but important step toward endowing the pervasive fiber-optics technology with unprecedented metasurface-enabled light-manipulation capabilities. From one side, this may dramatically increase the insofar quite limited impact and applicability of optical metasurfaces in real-world scenarios. From the viewpoint of the emerging lab-on-fiber paradigm, it represents an enabling factor with potentially disruptive implications, which significantly broadens the possible functionalities and application perspectives.

Within this framework, possible direct applications may include active beam profilers, spatial light modulators and fiber-optic tweezers. MT-based ‘flat-optics’ may also open up new venues in biomedical imaging, including scanning near-field optical microscopy and *in vivo* single molecule imaging. Also of great interest is the exploration of metasurface-based analog computing (along the lines of Silva *et al.*^32^), as well as tunability/reconfigurability mechanisms (e.g., electro-optic, magneto-optic, fluid-based) for the design of novel active, reconfigurable optical switches and frequency-agile nanodevices. In this context, the exploration of more efficient (e.g., dielectric-based) metasurface implementations is also of great interest, and is currently being pursued.

Finally, the results of our prototype study indicate promising perspectives in sensing applications. Especially, we found that the metasurface-induced phase distribution may affect the surface sensitivity in ways unexplored in the past. This brings about additional degrees of freedom and sophistication in the design and optimization of nanoplasmonic label-free chemical and biological sensors. We are currently working on the optimization of the phase distribution for maximizing the surface sensitivity, as well as on gaining a deeper insight of the role and interplay of the underpinning phenomena.

## Author contributions

VG and ACus conceived the experiment. MP and GC carried out the design and numerical simulations. AM, ACre, EE and VLF fabricated the prototypes. MC performed the experimental characterization and processed the measurement data. ACut, VG, ACus supervised the study. MP, VG and ACus wrote the manuscript, with inputs from all other authors.

## Figures and Tables

**Table 1:** Design parameters of the MT prototypes

	*ΔΦ*	*l*_x_(μm)	*N*	*Λ*_x_(μm)	*A*_p_(μm^2^)	*γ*_x_(rad cm^-1^)	*θ*_t_(^°^)	(*L*_1_,*L*_2_)(nm)
MT_1_	*π*/4	1	8	8	20 × 20	7854	11.2	(565, 365); (440, 320); (385, 240); (350, 100)
MT_2_	*π*/4	0.7	8	5.6	14 × 20	11 220	16.2	(565, 365); (440, 320); (385, 240); (350, 100)
MT_3_	*π*/3	0.7	6	4.2	14 × 20	14 960	21.8	(560, 410); (420, 340); (385, 190)
MT_4_	*π*/3	1	6	6	20 × 20	10 472	15.1	(560, 410); (420, 340); (385, 190)
MT_5_	*π*	0.53	2	1.06	15 × 15	59 275	–	(400, 120)

Only the sidelengths *L*_1_ and *L*_2_ of the nanoholes in the first half of the supercell are given, as the elements in the second half are obtained by a rotation of 90°. *A*_*p*_ denotes the extent of the patterned area. For all prototypes, the operational wavelength is *λ* = 1.56 μm, the gold-layer thickness is *h* = 50 nm, and the period along the *y*-direction is *Λ*_y_ = *l*_y_ = 1 μm.

**Table 2:** Numerically estimated efficiency (i.e., fraction of incident power transferred to the anomalous beam) for the four beam-steering MT prototypes

	Efficiency (%)
MT_1_	12
MT_2_	8
MT_3_	7
MT_4_	12

Parameters are given in Table 1, and details on the calculation are provided in the Supplementary Information.

## Supplementary Material

Supplementary Information

Supplementary Movie 1 Caption
