# Supplementary material for: Optical fiber meta-tips
Source: Light Sci Appl. 2017 Mar 10;6(3):e16226–. doi: 10.1038/lsa.2016.226 (PMC6062173; doi:10.1038/lsa.2016.226)
Supplement: Supplementary Information [file lsa2016226x1.docx]

**Optical fiber meta-tips: Supplementary information**

Maria Principe^1,2,+^, Marco Consales^1,+^, Alberto Micco^1^, Alessio Crescitelli^3^, Giuseppe

Castaldi^2^, Emanuela Esposito^3^, Vera La Ferrara^4^, Antonello Cutolo^1^, Vincenzo Galdi^2,*^,

and Andrea Cusano^1,*^

^1^Optoelectronic Division, Department of Engineering, University of Sannio, I-82100 Benevento, Italy

^2^Waves Group, Department of Engineering, University of Sannio, I-82100 Benevento, Italy

^3^Institute for Microelectronics and Microsystems, National Research Council, I-80131, Napoli, Italy

^4^UTTP-MDB, Materials and devices, ENEA — Portici Research Center, I-80055 Portici (NA), Italy

^*^Correspondence and requests for materials should be addressed to V.G. (vgaldi@unisannio.it) or A.C.

(a.cusano@unisannio.it)

^+^these authors contributed equally to this work

**ABSTRACT**

This document contains additional details on the design, fabrication and experimental characterization, as well as supplementary results. Newly introduced equations are labeled with the prefix “S”, whereas newly introduced figures and tables are referred to as “Supplementary” and are labeled with the prefix “S”; all others pertain to the main text. All references are intended as local, and those already utilized in the main text are repeated.

**SUPPLEMENTARY MATERIALS AND METHODS**

**Design procedure and numerical modeling**

Our metasurface design relies on a preliminary numerical study of the transmission coefficient pertaining toa 2-D periodic array of *identical* rectangular nanoholes. These numerical simulations are carried out by means of the RF module available in the finite-element-based commercial software COMSOL Multiphysics (www.comsol.com).

The periodic array is placed at the interface between two homogeneous halfspaces made of silica and air. We consider a square unit-cell with sidelength$l_{x}=l_{y}=1 \mu\text{m}$which, at the operational wavelength of interest $\lambda=1.56 \mu\text{m}$, ensures that only the fundamental (zeroth) diffraction order propagates in the two regions.The unit cell is terminated by periodic Bloch-type boundary conditions along the $x$- and $y$- directions. Port-type terminations are instead assumed along the incidence direction $z$, at a distance $\lambda_{a}$ (i.e., ambient wavelength) from the array, and matched with the zeroth-order modes for both $x$- and $y$-polarized illuminations. An adaptive meshing is utilized, with maximum element size of $\lambda_{a}/10$ in the uniform dielectric regions, and of 15 nm in the air regions of the array; for the gold layer, a minimum number of two elements per skin depth ($\delta_{s}=23.5 \text{nm}$ at $\lambda=1.56 \mu\text{m}$) is employed.

**Supplementary Figure S1 |Design look-up maps.a, b** Numerically-computed phase distributions of co-polar and cross-polar, respectively, transmission coefficient, as a function of nanohole dimensions, assuming an infinite square array with period $l_{x}=l_{y}=1 \mu\text{m}$ with normally-incident, $x$-polarized plane-wave illumination at $\lambda=1.56 \mu\text{m}$. For better visualization, the phase distributions are “unwrapped”.**c, d** Corresponding magnitude maps. As a reference, a representative equi-magnitude contour ($\left|T_{xy}\right|=0.3$) is superposed (dashed-magenta curve) to the cross-polar phase map in panel b.

Overall, this results in about 700,000 degrees of freedom. The MUMPS solver is utilized, with default parameters.

By varying the nanoholesidelengths$L_{1}$ and $L_{2}$ (see Figure 1c) around their resonance values, and illuminating the array with a normally-incident$x$-polarized plane wave at the operational wavelength, we obtain the “look-up” maps shown in Supplementary Figure S1. More specifically, the maps are calculated on a $14\times14$ grid of ($L_{1},L_{2}$) values, and bi-linear interpolation is used for a finer sampling. The transmission coefficients are computed as the scattering parameter pertaining to co- and cross-polarized incident and transmitted zeroth-order modes.

It can be observed that, different from the co-polarized case (Supplementary Figure S1a), the phase map pertaining to the cross-polarized component (Supplementary Figure S1b) spans a full $2\pi$ range, which is a necessary condition for designing an arbitrary phase profile. In spite of the inherent efficiency limits in the underlying polarization-conversion mechanism, the desired phase span can be attained for moderate values of the transmission-coefficient magnitude, as shown by the reference equi-magnitude contour ($\left|T_{xy}\right|=0.3$) superposed on the cross-polar phase map.

Based on the look-up maps, we can select the nanohole dimensions so as to synthesize a desired phase distribution of the cross-polar transmission coefficient. More specifically, given the targeted phase-gradient $\gamma_{x}$ and an even number $N$ of elements in a super cell, we initialize the synthesis procedure by choosing the dimensions $\left(L_{1}^{(1)},L_{2}^{(1)}\right)$ of the first element, yielding a phase $\Phi_{1}$ of the cross-polar transmission coefficient$T_{xy}$. We then proceed with the subsequent elements by minimizing the cost function

$$J\left(L_{1}^{(n)},L_{2}^{(n)}\right)=\left\{\arg\left[T_{xy}\left(L_{1}^{(n)},L_{2}^{(n)}\right)\right]-(\Phi_{n}+\Phi_{1})\right\}^{2}+\beta\left[\left|T_{xy}\left(L_{1}^{(n)},L_{2}^{(n)}\right)\right|-T_{xy}^{(max)}\right]^{2}, n=2,3,\ldots,N/2,$$

where$\Phi_{n}$ denotes the desired phase value, $\arg$ is the argument, $\beta\in[0,1]$ is a weight coefficient, and $T_{xy}^{(max)}$ is the maximum value of $\left|T_{xy}\right|$ that allows a full $2\pi$ phase excursion within the $(L_{1}^{(n)},L_{2}^{(n)})$-space. Here, and henceforth the principal value of the phase (within the $[0,2\pi]$ range) is assumed. The cost function is minimized via a standard Nelder-Meald (simplex) unconstrained optimization algorithm. The parameters $\Phi_{1}$, $\beta$ and $T_{xy}^{(max)}$ are chosen heuristically, via trial-and-error.

The remaining $N/2$ elements can be obtained via a rotation of 90° in the $x-y$ plane, which provides a $\pi$phase-shift on the cross-polar transmission coefficient.^1^Clearly, the number of free parameters is not sufficient to control both polarization components. Nonetheless, this procedure allows us to obtain a co-polar transmission coefficient with sufficiently uniform magnitude and phase distributions over the designed supercells (see Figure 2). Additional degrees of freedom, e.g., the rotation angle of the nanoholes, could be used to attain better control on both polarization components.

The phase gradient can be increased by either reducing $l_{x}$, or by increasing the inter-element phase difference$\Delta\Phi$. However, some limitations exist, since $l_{x}$ should be large enough to limit the inter-element coupling effects, and $\Delta\Phi$ can be at most equal to $\pi$ in order to guarantee the correct reconstruction of the linear phase profile. The synthesis results summarized in Table 1 implement various combinations of these two parameters.

**Supplementary Figure S2 |Results from the design procedure (MT_2_ and MT_4_).a, b** Numerically-synthesized phase and magnitude distributions, respectively, of the transmission coefficient pertaining to the single nanoholes in the supercell (shown on top) of the MT_2_ design (with parameters as given in Table 1), for the co-polarized (blue square markers) and cross-polarized (red circle markers) components, assuming an infinite periodic array of period $l_{x}=l_{y}=1 \mu\text{m}$, under normally-incident $x$-polarized plane-wave illumination at $\lambda=1.56 \mu\text{m}$. Element #1 is chosen as phase reference. Continuous curves are guides to the eye only. **c, d** Same as above, but for MT_4_ design.

We verified that the look-up maps in Supplementary Figure S1, calculated for a specific inter-element spacing ($l_{x}=l_{y}=1 \mu\text{m}$), could also be utilized for designs featuring slightly smaller values of $l_{x}$ (e.g., MT_2_ and MT_3_), yielding only slight distortions. Supplementary Figure S2 shows the phase and magnitude profiles pertaining to the MT_2_and MT_4_ designs (not shown in the main text for brevity). We highlight that, for the MT_5_ design, any value of the nanoholesidelengths would produce the same phase gradient. However, the chosen design was found to provide a good trade-off between small size (so as to reduce $l_{x}$) and high cross-polar transmission coefficient.

The far-field intensity profiles in Figure 4 (as well as Supplementary Figure S7below) are computed in two steps. First, we compute the transmitted field pertaining to a MT of finite width ~$20\mu\text{m}$ along the $x$-direction, and assumed as infinitely-periodic in the $y$-direction, illuminated by a 1-D Gaussian beam with waist size of $5\mu\text{m}$ impinging from the silica region (so as to partially mimic the modal field of the SMF-28 optical fiber).The structure is terminated by Bloch-type periodic boundary conditions along the $y$-direction, and by *ad hoc*perfectly matched layers (PML) in the remaining directions. The above assumptions, which neglect the non-uniform polarization distribution (along the $y$-direction) of a realistic 2-D beam fiber-optic illumination, are instrumental to maintain the computational burden within affordable limits, while still providing a good estimate of the illumination-tapering effects.

The near-field distribution is computed by means of COMSOL Multiphysics, with the simulated region extending up to a distance of $\lambda_{a}/2$ in air and $\lambda_{a}/5$ in the fiber region, in order to guarantee the computational affordability. Once again, an adaptive meshing is employed, with maximum element size of $\lambda_{a}/8$ in the uniform dielectric regions, 50nm in the air regions of the array, and a minimum number two element per skin-depth in the gold layer,resulting in about 4.5 million degrees of freedom. The PARDISO solver is utilized, with default parameters.As a second step, the computed near-field is propagated to the far-field region using well-known formulae for the radiation from planar apertures (e.g., Sec. 4.1 in Ref. 2). In order to account for the lack of polarization control in the measurements shown in Figure 4 (as well as Supplementary Figure S7 below), the far-field intensity profiles are averaged over the two limiting cases of$x$- and $y$-polarized incidence. Results are normalized with respect to the maximum values.

From the same numerical simulations, we also estimate the efficiency of the designed MT prototypes, i.e., the fraction of incident power that gets transferred to the anomalous beam. To this aim, assuming an$x$-polarized incidence, we compute the flux of the Pointing vector (real-part) associated with the cross-polarized transmitted field through a planar surface at a distance $\lambda/2$ from the metasurface. We then obtain the efficiency by normalizing this quantity by the same flux pertaining to the incident field only, calculated in the absence of the metasurface, and by truncating the fiber region with a PML.

The field maps shown in Figures7−9are also computed via COMSOL Multiphysics by assuming a periodic 2-D array under normally-incident$x$-polarized plane-wave illumination. Once again, Bloch-type periodic boundary conditions are assumed along the $x$- and $y$-directions, with *ad hoc* PML terminations along the $z$-direction. In this case, the simulated region extends up to a distance of $\lambda_{a}$ in air and $\lambda_{a}/4$ in the fiber region, with maximum mesh-element size of $\lambda_{a}/15$ in the uniform dielectric regions and of 20nm in the air regions of the array, and a minimum number two element per skin-depth in the gold layer (resulting in about 2 million degrees of freedom). Also in this case, the MUMPS solver is utilized. Results are normalized with respect to the incident-field amplitude.

For better computational affordability, the reflectivity spectra for these configurations are computed by means of a public-domain numerical code (sourceforge.net/projects/rcwa-2d/files/) that implements a 2-D rigorous coupled wave analysis (RCWA).^3^ Once again, a normally-incident$x$-polarized plane-wave illumination is assumed. Convergence is achieved by using $51\times51$ modes. To realistically simulate the experimental reflection setup, the reflectivity pertaining to the zero-th diffraction order is considered, corresponding to light traveling back along the fiber axis. Moreover, in order to roughly mimic a realistic deposition process, the SiO_x_ overlay is assumed as conformal to the MT surface (i.e., filling the nanoholes and covering the unpatterned gold regions).

**Supplementary Figure S3 |Morphological characterization.a, b** AFM topographic images (2-D top view and 3D perspective view, respectively) pertaining to MT_3_ sample after the FIB milling of the gold layer. The rectangular shaded area delimits a set of 42 nanoholes (six supercell replicas) considered in the statistical study (see Supplementary Table S1).

**Prototype fabrication**

The fabricated samples are affected by imperfections and tolerances inherent to the fabrication process. In order to investigate the geometrical deviation of the nanohole shapes with respect to the nominal design, we carried out an extensive morphological characterization of the fabricated samples. For instance, with reference to the MT_3_ sample, Supplementary Figure S3shows the atomic-force-microscope (AFM) topographic images of the fiber tip after the FIB patterning. Multiple measurements on the nanoholesidelengths ($L_{1},L_{2}$) are performed on the image, with results summarized in Supplementary Table S1. More specifically, a set of 42 nanoholes is considered (rectangular shaded area in Supplementary Figure S3a), which basically comprises seven replicas (rows) of the six-elementsupercell (see Table 1 and Figure 2c inset). It can be observed that the measured values are always slightly larger than the nominal-design ones, with relative errors within the range 4-9%. This is attributable to the Gaussian shape of the ion beam, which creates a smoothed profile of the nanoholes along the edges. Another fabrication-related issue is the gallium ion doping of the silica substrate that occurs during the patterning,^4^ which may induce unmodeled variations in the fiber refractive index.

**Supplementary Table S1 |Morphological characterization.**Statistics of AFM measurements of the nanoholesidelengths ($L_{1},L_{2}$) pertaining to a set of 42 elements (shaded rectangular area in Supplementary Figure S3a) in MT_3_ sample.

|  | element #1 | | element #2 | | element #3 | | element #4 | | element #5 | | element #6 | |
| --- | --- | --- | --- | --- | --- | --- | --- | --- | --- | --- | --- | --- |
| row # | $L_{1}\left(\text{nm}\right)$ | $L_{2}\left(\text{nm}\right)$ | $L_{1}\left(\text{nm}\right)$ | $L_{2}\left(\text{nm}\right)$ | $L_{1}\left(\text{nm}\right)$ | $L_{2}\left(\text{nm}\right)$ | $L_{1}\left(\text{nm}\right)$ | $L_{2}\left(\text{nm}\right)$ | $L_{1}\left(\text{nm}\right)$ | $L_{2}\left(\text{nm}\right)$ | $L_{1}\left(\text{nm}\right)$ | $L_{2}\left(\text{nm}\right)$ |
| 1 | 598 | 438 | 450 | 362 | 410 | 218 | 439 | 609 | 359 | 445 | 202 | 411 |
| 2 | 592 | 445 | 456 | 362 | 404 | 218 | 433 | 595 | 360 | 431 | 205 | 406 |
| 3 | 598 | 431 | 444 | 355 | 404 | 211 | 439 | 589 | 365 | 438 | 205 | 411 |
| 4 | 587 | 424 | 461 | 369 | 410 | 205 | 444 | 595 | 360 | 438 | 194 | 404 |
| 5 | 592 | 431 | 456 | 369 | 404 | 191 | 439 | 589 | 358 | 424 | 202 | 400 |
| 6 | 592 | 445 | 456 | 355 | 404 | 205 | 444 | 589 | 353 | 445 | 205 | 402 |
| 7 | 587 | 431 | 444 | 362 | 416 | 205 | 439 | 589 | 358 | 445 | 210 | 392 |
| mean (nm) | 592 | 435 | 452 | 362 | 407 | 208 | 440 | 594 | 359 | 438 | 203 | 404 |
| std.dev. (nm) | 4 | 8 | 7 | 6 | 5 | 9 | 4 | 7 | 4 | 8 | 5 | 7 |
| nominal (nm) | 560 | 410 | 420 | 340 | 385 | 190 | 410 | 560 | 340 | 420 | 190 | 385 |
| abs.err. (nm) | 32 | 25 | 32 | 22 | 22 | 18 | 30 | 34 | 19 | 18 | 13 | 19 |
| rel.err. (%) | 6 | 6 | 8 | 6 | 6 | 9 | 7 | 6 | 6 | 4 | 7 | 5 |

Overall, the fairly good agreement observed between measurements and numerical simulations seems to indicate a substantial robustness of the designs with respect to these fabrication-related tolerances and imperfections.

In view of the unconventional nature of the fiber-tip substrate for the electron beam evaporation system, it is important to verify the gold layer thickness. To this aim, after the deposition, a KrF pulsed excimer laser (Optec LB1000, operating at a wavelength of 248nm) is used to mill a rectangular hole into the gold layer. A 4mJ energy is used, with attenuation of 30%, repetition rate of 50Hz, and exposure time of 1s. The thickness measurement is performed by means of an AFM (Agilent Technologies 5420), using a proper fiber holder. The measurement is made in contact mode with a Nanosensors PPP-CONT tip, at a speed of 1ln/s, and yields a thickness value of about 51.3nm, very close to the nominal value of 50nm.

In connection with the SiO_x_ overlay deposited for the surface-sensitivity characterization,it is well known that, in the PECVD process, the reaction can be in the mass-transport-limited regime, where the rate is dependent on the supply of the gas to the surface. Fluid dynamics in the chamber plays a major role in the deposition rate, and the presence of the fiber can cause turbulence around the tip. Therefore, a measurement of the overlay thickness on the fiber tip is crucial.Similar to the previous measurement, a rectangular slot is milled on the fiber tip, after the oxide film deposition, by exposure to an excimer laser. In this case, a 6mJ energy is used, with attenuation of 60%, repetition rate of 150Hz, and exposure time of 1s. Subsequently, the thickness of the oxide layer is inspected via an AFM measurement, which yields a value of about 40nm.

**Supplementary Figure S4 |Schematic of the experimental setup for far-field characterization without polarization control.** For the polarization measurements, a fiber polarization controller is connected at the output of the tunable laser, and a linear polarizer is mounted on a continuous rotation mount positioned right before the vidicon camera.

**Experimental characterization**

Supplementary Figure S4schematically illustrates the far-field experimental setup.To facilitate direct comparison with the numerical predictions (cf. Figure 4), the acquired field-intensity maps are suitably normalized (with respect to the maximum values), rotated and registered so as to have the peak corresponding to the ordinary beam at coordinates $x=y=0$. The transmission angles are estimated via a differential scheme (see Supplementary Figure S5), by measuring the separation between the two main peaks at two different distances from the receiving window of the vidicon camera, whose difference is accurately controlled via the micro-positioning system.We therefore obtain the estimate

$$\theta_{t}^{(exp)}=\arctan\left(\frac{d_{2}-d_{1}}{\Delta}\right).$$

By taking into account the resolution of the micrometer positioning system as well as the pixel size of the acquired images, we estimate a$\pm0.6^{\circ}$ uncertainty.

**Supplementary Figure S5 |Transmission-angle measurement.**Illustration (not in scale) of the differential measurement scheme [see Equation ].

For the polarization measurements (see Figure 5 and Supplementary Movie 1), the position of the MT sample on the plastic holder is judiciously adjusted so as to align the phase-gradient direction (*x*-axis in the assumed reference system; see Figure 1) parallel to the vidicon camera horizontal axis. By suitably acting on the fiber polarization controller and the linear polarizer, the incident polarization on the MT is aligned with the *y*-axis, in such a way the ordinary and anomalous beams are fiducially polarized along the *y*- and *x*- directions, respectively. The measurements in Figure 5 and Supplementary Movie 1 are performed by suitably rotating the polarizer so as to select specific linear-polarization states of the transmitted field.

**Supplementary Figure S6 |Schematic of the experimental setup for surface-sensitivity characterization.**

Supplementary Figure S6 shows a schematic of the surface-sensitivity experimental setup. To minimize the influence of the light-source spectral features, as well as of the losses introduced by the optical chain, the acquired spectrum is normalized by that of a reference mirror fabricated by depositing a 160 nm gold layer on the facet of a standard single-mode fiber. To filter out random noise, the normalized spectrum is finally smoothed via the filtfilt function (zero-phase digital filtering) available in Matlab ([www.mathworks.com](http://www.mathworks.com)).

**SUPPLEMENTARY RESULTS**

With reference to the MT_3_ sample, Supplementary Movie 1 shows the evolution of the measured field map, by assuming a *y*-polarized incidence and gradually changing (with 5° step) the transmitted linear polarization state by 90°. Going from the co-polarized to the cross-polarized state, and in accord with the theoretical prediction, we observe the gradual disappearing of the ordinary beam accompanied by the appearance of the anomalous beam.

Supplementary Figure S7 shows the results of the far-field characterization (without polarization control) pertaining to the MT_2_ and MT_4_ samples. Overall, results are qualitatively similar to those observed in connection with the MT_1_ and MT_3_ prototypes (cf. Figure 4). In this case, transmission angles $\theta_{t}^{(exp)}=15.3^{\circ}$ (for MT_2_) and $\theta_{t}^{(exp)}=14.2^{\circ}$ (for MT_4_) are measured, once again in good agreement with the theoretical predictions (see Table 1). Measurements with polarization control yield results qualitatively similar to those in Figure 5 and Supplementary Movie 1 pertaining to the MT_3_ sample.

**Supplementary Figure S7 | Far-field characterization (MT_2_ and MT_4_) without polarization control.a**Simulated electric-field intensity profiles (at $z=8 \text{mm}$ and $y=0$) of the ordinary and anomalous beams (blue and red curves, respectively), for MT_2_ sample (with parameters as given in Table 1). Resultsare obtained by averaging the co-polar and cross-polar responses, respectively, undernormally-incident$x$- and $y$-polarized illuminations at $\lambda=1.56 \mu\text{m}$.**a** Measured field-intensity map at $z=8 \text{mm}$. **c**Transverse cuts at$y=0$comparing the measured (black-solid curve) and simulated (magenta-dashed curve) results.Numerical results are obtained by averaging the total electric-field intensities for normally-incident$x$- and $y$-polarized illuminations. The structure is considered as infinitely-periodic along $y$, whereas, along the $x$-direction, a finite-size$\sim20 \mu\text{m}$ is assumed, together with a Gaussian-beam taper (with waist size of $5 \mu\text{m}$)in the illumination. **d, e, f** Same as above, but for MT_4_ sample.

**REFERENCES**

1 Yu N, Genevet P, Aieta F, Kats MA, Blanchard R*et al.* Flat optics: Controlling wavefronts with optical antenna metasurfaces. *IEEE JSelect Topics Quantum Electron* 2013;**19**:4700423.

2 Collin RE.*Antennas and Radiowave Propagation*. McGraw-Hill; 1985.

3 Moharam MG, Gaylord TK, Grann EB, Pommet DA. Formulation for stable and efficient implementation of the rigorous coupled-wave analysis of binary gratings. *J Opt Soc Am A*1995;**12**:1068-1076.

4 Micco A, Ricciardi A, Pisco M, La Ferrara V, Cusano A. Optical fiber tip templating using direct focused ion beam milling. *Sci Rep* 2015;**5**: 15935.
