# Supplementary material for: Optical fiber meta-tips
Source: Light Sci Appl. 2017 Mar 10;6(3):e16226–. doi: 10.1038/lsa.2016.226 (PMC6062173; doi:10.1038/lsa.2016.226)
Supplement: Supplementary Movie 1 Caption [file lsa2016226x2.docx]

**Supplementary Movie 1** **| Evolution of the measured field map pertaining to MT_3_ sample.** The incident beam is *y*-polarized (red arrow), and the transmitted linear polarization state is gradually rotated (5° step) by 90°, as indicated by the yellow arrow. Also shown are the supercell geometry (upper-left corner) and the expected polarizations of the ordinary and anomalous beams (green and cyan arrow, respectively). The intensity is represented in greyscale, with white corresponding to highest values. Going from the co-polarized to the cross-polarized state, the ordinary beam gradually disappears, while the anomalous beam gradually appears.
